# Differences between immunodeficient mice generated by classical gene targeting and CRISPR/Cas9-mediated gene knockout

**DOI:** 10.1007/s11248-018-0069-y

**Published:** 2018-03-28

**Authors:** Jae Hoon Lee, Jong-Hyung Park, Tae-Wook Nam, Sun-Min Seo, Jun-Young Kim, Han-Kyul Lee, Jong Hyun Han, Song Yi Park, Yang-Kyu Choi, Han-Woong Lee

**Affiliations:** 1 Department of Biochemistry, College of Life Science and Biotechnology, Laboratory Animal Research Center, Yonsei University, Seoul, 03722 Republic of Korea; 2 Department of Laboratory Animal Medicine, College of Veterinary Medicine, Konkuk University, Seoul, 05029 Republic of Korea

**Keywords:** *Il2rg*, *Prkdc*, *Rag2*, CRISPR/Cas9, Immunodeficient mouse models

## Abstract

Immunodeficient mice are widely used for pre-clinical studies to understand various human diseases. Here, we report the generation of four immunodeficient mouse models using CRISPR/Cas9 system without inserting any foreign gene sequences such as Neo^R^ cassettes and their characterization. By eliminating any possible effects of adding a Neo^R^ cassette, our mouse models may allow us to better elucidate the in vivo functions of each gene. Our FVB-*Rag2*^−/−^, B6-*Rag2*^−/−^, and BALB/c-*Prkdc*^−/−^ mice showed phenotypes similar to those of the earlier immunodeficient mouse models, including a lack of mature B cells and T cells and an increase in the number of CD45^+^DX-5^+^ natural killer cells. However, B6-*Il2rg*^−/−^ mice had a unique phenotype, with a lack of mature B cells, increased number of T cells, and decreased number of natural killer cells. Additionally, serum immunoglobulin levels in all four immunodeficient mouse models were significantly reduced when compared to those in wild-type mice with the exception of IgM in B6-*Il2r*g^−/−^ mice. These results indicate that our immunodeficient mouse models are a robust tool for in vivo studies of the immune system and will provide new insights into the variation in phenotypic outcomes resulting from different gene-targeting methodologies.

**Electronic supplementary material:**

The online version of this article (10.1007/s11248-018-0069-y) contains supplementary material, which is available to authorized users.

## Introduction

Since severe combined immunodeficiency (SCID) mice lacking functional B and T lymphocytes were first spontaneously discovered in a colony of C.B-17 mice (Bosma et al. 1983), many immunodeficient mice have served as invaluable model organisms in both biological and clinical studies (Ito et al. 2002; Ohbo et al. 1996; Shinkai et al. 1992). Among them, *Rag2*-deficient mice fail to generate mature B and T cells due to the impairment of the V(D)J rearrangements that are crucial to initiating the development of functional immunoglobulin (Ig) and T cell receptors (TCR) (Shinkai et al. 1992). Similarly, deficiency of a DNA-dependent protein kinase catalytic subunit (DNA-PKcs; encoded by *Prkdc* gene) causes a SCID phenotype that is characterized by an absence of functional B and T cells, lymphopenia, hypogammaglobulinemia, but a normal hematopoietic microenvironment (Buckley et al. 1997; Puck et al. 1997). Therefore, SCID mice are acceptable for both allogeneic and xenogeneic cell transfer experiments. Unlike other immunodeficient mice lacking B and T cell development, however, the absence of IL2rγ in mice disrupts not only B and T cells but also natural killer (NK) cells (Belizario 2009). This permits the engraftment with human hematopoietic cells to establish a human immune system in this mouse model. Humanized mice containing human cells or tissues are becoming increasingly important as animal models for studying basic and applied human diseases (Honeycutt et al. 2015; Ito et al. 2012; Walsh et al. 2017). To improve the utility of immunodeficient mouse models, we generated four different models using the CRISPR/Cas9 system and evaluated whether different background strains or targeted genes affect the immunodeficiency phenotypes.

The CRISPR/Cas9 system can generate improved genetically modified mouse models, because it does not require the inclusion of a drug resistance marker such as a PGK-neo cassette which is required to generate ES cell-based knockout mice. The Cas9 derived from *Streptococcus pyogenes* recognizes 5′-NGG-3′ as the protospacer adjacent motif (PAM), and the sgRNA consists of a sequence complementary to the 20 nucleotides upstream of the PAM to identify the target site (Wright et al. 2016). The double-strand breaks (DSBs) generated by the Cas9/sgRNA ribonucleoprotein complex lead to either non-homologous end joining (NHEJ) or homologous recombination (HR) repair—mechanisms that are used to generate knockout and knock-in mice, respectively (Lieber 2010; Vasquez et al. 2001). Without a template for HR-mediated DNA repair, the DSB will be repaired through the error-prone NHEJ pathway, forming insertion and deletion (indels) mutations in the target gene that may lead to frameshift mutations (Lieber 2010). Loss of gene function is often induced by premature stop codons downstream of the frameshift mutation using the CRISPR/Cas9 system approach. Importantly, our models lack PGK-neo cassettes that are usually included in existing knockout mouse models. We evaluated whether CRISPR/Cas9-mediated knockout mice exhibit phenotypes different from those of previous immunodeficient mouse models that were generated by classical gene targeting using ES cells.

## Materials and methods

### Animals and ethics statement

BALB/cAnNTac (BALB/c), C57BL/6JBomTac (B6), FVB/NTac (FVB), and IcrTac:ICR (ICR) mice were purchased from Taconic Biosciences (Dae Han Biolink Co., Ltd., Chungbuk, Republic of Korea). All mice were housed in the specific pathogen-free (SPF) facility of the Yonsei Laboratory Animal Research Center. All efforts were made to minimize animal suffering, and all animal experiments were conducted in accordance with the Korean Food and Drug Administration (KFDA) guidelines. Experimental protocols were reviewed thoroughly and approved by the Institutional Animal Care and Use Committees (IACUC) at Yonsei University (Permit Number: 201506-322-02). All immunodeficient mouse models presented in this study will be made readily available to the research community.

### Preparation of *CRISPR/Cas9* mRNA

The mMESSAGE mMACHINE^®^ T7 Ultra kit (Ambion) was used to obtain the *Cas9* mRNA, which was diluted in diethyl pyrocarbonate (Sigma)-treated injection buffer (0.25 mM EDTA, 10 mM Tris, pH 7.4) to obtain the working concentration. Additionally, the MEGAshortscript T7 Transcription kit (Ambion) was used to synthesize sgRNAs from PCR-generated templates. Plasmids encoding *S. pyogenes* Cas9 (SpCas9) protein (Cho et al. 2013) were obtained from ToolGen, Inc. (Seoul, Republic of Korea).

### Microinjection

To generate immunodeficient mice using CRISPR/Cas9, microinjection of fertilized embryos was performed: initially, 6–8-week-old BALB/c, B6, and FVB mice were super-ovulated by intra-peritoneal injections of 5 IU pregnant mare serum gonadotropin (Sigma) and 5 IU human chorionic gonadotropin (Sigma) at 48-h intervals. The fertilized embryos were then collected from the super-ovulated mice crossed with stud males. A mixture of 50 ng/μL of *Cas9* mRNA and 250 ng/μL of sgRNA was microinjected into the cytoplasm of zygotes, using a piezo-driven manipulator (Prime Tech) to induce mutations, and the resulting embryos were transferred into the oviducts of ICR pseudo-pregnant foster mothers to produce live mice.

### Founder screening and genotyping PCR

To screen founder mice for endonuclease-mediated mutations such as indels, PAGE-PCR assays were performed using genomic DNA samples from tail biopsies (Zhu et al. 2014). In brief, the genomic regions spanning the sgRNA target site were amplified by PCR. By simply denaturing and annealing, PCR products containing a mixture of mutant and wild-type alleles form heteroduplex DNA and homoduplex DNA as described previously (Zhu et al. 2014). Since heteroduplex DNA migrates slower than homoduplex DNA under a nondenaturing condition, PCR products carrying indels were analyzed by acrylamide gel electrophoresis. The PCR products from founder mice were also cloned using the T-Blunt PCR Cloning Kit (SolGent Co., Ltd., Republic of Korea) for sequence analysis, and the mutations were identified by direct sequencing analysis (Cosmobiotech Co., Ltd., Republic of Korea). Those that exhibited the earliest premature stop codons were selected as founder mice.

### Antibodies and flow cytometry

Eight-week-old mice were sacrificed and single cells from the spleen, thymus, and bone marrow were prepared for flow cytometry analysis. To obtain single-cell suspensions, tissues were cut into small pieces with scissors and passed through a 70-μm cell strainer (BD Biosciences, Franklin Lakes, NJ, USA) by pressing with a plunger. The remaining red blood cells were eliminated by using 1 × RBC lysis buffer (eBioscience, San Diego, CA, USA). Prepared cells were stained with FITC-anti-B220 (RA3-6B2; BD Biosciences), PerCP/Cy5.5-anti-IgM (RMM-1; BioLegend, San Diego, CA, USA), APC-anti-CD3ε (145-2C11; BD Biosciences), PE-anti-TCR (H57-597; Santa Cruz Biotechnology, Santa Cruz, TX, USA), Alexa Fluor 488-anti-CD4 (RM4-5; BD Biosciences), APC-anti-CD8α (53-6.7; BioLegend), PE-anti-CD45 (30-F11; BD Biosciences), and APC-anti-CD49b (DX-5; BD Biosciences) antibodies. At least 10,000 live cells were analyzed with the FACSCalibur system (BD Biosciences).

### Enzyme-linked immunosorbent assay (ELISA)

Serum samples were collected from the caudal veins of both homozygous mutant and wild-type mice and then separated in a refrigerated centrifuge at 4 °C. Serum immunoglobulin levels of mice were measured using the mouse Ready-SET-Go Kits plate (eBioscience, San Diego, CA, USA) following the manufacturer’s protocol. Briefly, 96-well ELISA plates were coated with the purified anti-mouse IgG1, IgG2a, IgG2b, IgG3, IgA, or IgM monoclonal antibody in coating buffer at 4 °C overnight and then blocked with diluted assay buffer for 2 h at room temperature, followed by washing two times. Serial dilutions of pre-titrated standards (IgG1, IgG2a, IgG2b, IgG3, IgA, and IgM) and 50 μL of diluted mouse sera were added and the plates incubated for 1 h at room temperature. After washing four times, avidin-horseradish peroxidase-conjugated antibodies were added and the plates incubated again for 1 h at room temperature. After another four washes, the samples were incubated with the substrate 3,3,5,5—tetramethylbenzidine (TMB) for 15 min followed by the addition of TMB stop solutions. The absorbance was read in a Tecan spectrometer plate reader (Männedorf, Switzerland) at 450 nm with a background subtraction of 570 nm.

### Immunohistochemical staining

Formalin-fixed, paraffin-embedded samples including the spleen, thymus, and lymph nodes, of 8-week-old homozygous null and wild-type mice were used for immunohistochemical staining. Anti-B220 (RA3-6B2; Abcam, Cambridge, UK) and anti-CD4 (EPR19514; Abcam) antibodies were used for B cell and T cell immunostaining, respectively.

### Statistical analysis

Statistically significant differences between groups were calculated using the unpaired t-test (GraphPad Software, Inc., La Jolla, USA). The results were considered statistically significant at *P* < 0.05, *P* < 0.01, or *P* < 0.001.

## Results

### Generation of immunodeficient mice carrying out-of-frame alleles of *Rag2, Prkdc,* or *Il2rg*

To generate immunodeficient mice carrying disruptions of the *Rag2, Prkdc*, or *Il2rg* genes, we used CRISPR/Cas9 system. Each sgRNA was designed to cleave downstream of the start codon of the genes of interest to induce frameshift mutations caused by non-homologous end joining (NHEJ)-mediated DNA repair. To avoid potential off-target effects of each sgRNA, we designed several sgRNAs using CRIPSR Design (http://crispr.mit.edu) and sgRNA Design Tool (https://portals.broadinstitute.org/gpp/public/analysis-tools/sgrna-design), and then intentionally chose the sgRNAs to recognize the specific sequence that contains no apparent homology with the mouse genome sequence. We directly injected *Cas9* mRNA with the target-specific sgRNA(s) into one-cell embryos and screened the mutants by PAGE-PCR assay using genomic DNAs obtained from the newborns. We successfully generated several mutant mice with high efficiency (Table S1) and selected founder mice (F0) harboring nucleotide excision leading to premature termination of each target protein (Fig. S1). These mutations introduced premature termination codons (PTCs) into the open reading frames of the target genes, leading to loss of gene function. All immunodeficient mouse strains were then validated in offspring derived from each founder mouse by sequence analysis. The homozygous knockout mice (F2) were generated from an intercross between F1 heterozygous knockout mice and confirmed by PCR genotyping (Fig. S1 and Table S2). Since mRNAs with PTCs are targeted by the nonsense-mediated decay (NMD) pathway, RT-PCR analysis showed significant reduction of each mRNA in the immune system tissues of the homozygous knockout mice compared with the wild-type mice, except for the thymus of B6-*Rag2*^−*/*−^ and BALB/c-*Prkdc*^−/−^ mice (Fig. S2).

### Immunophenotypes of *Rag2*^−/−^ mice

**Fig. 1:**
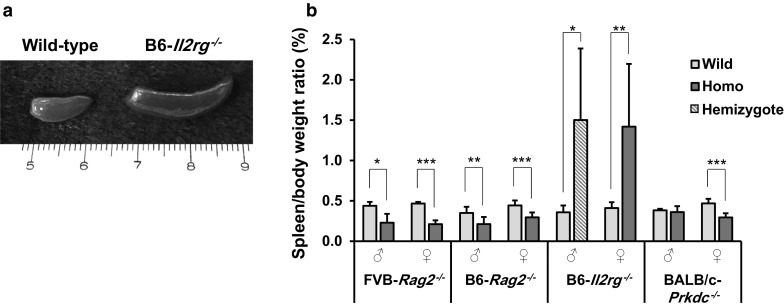
Spleen-to-body weight ratio in immunodeficient mice. Spleens of FVB-*Rag2*^−/−^, B6-*Rag2*^−/−^, and BALB/c-*Prkdc*^−/−^ mice were significantly smaller than those of wild-type mice. Unlike other mutant strains, B6-*Il2rg*^−/−^ mice showed severe splenomegaly. Each value represents the mean ± SD (n = 4–5 mice per group). *P < 0.05, **P < 0.01, and ***P < 0.001

**Fig. 2:**
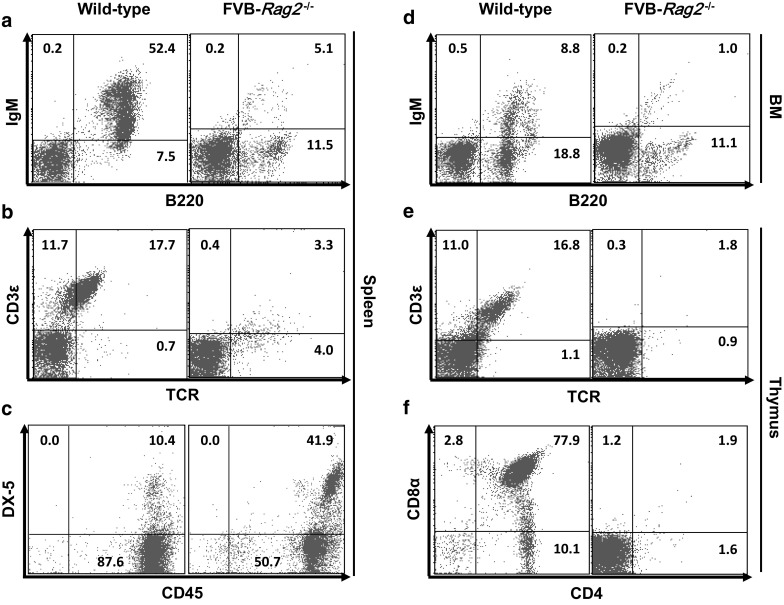
Flow cytometric analysis of lymphoid cells in 8-week-old FVB-*Rag2*^−/−^ mice. **a**–**c** analysis of B, T, and NK cells in the spleen. **d** Analysis of B cells in the bone marrow. **e**, **f** Analysis of T cells in the thymus. Each value represents the mean ± SD. (n = 10 mice per group)

**Fig. 3:**
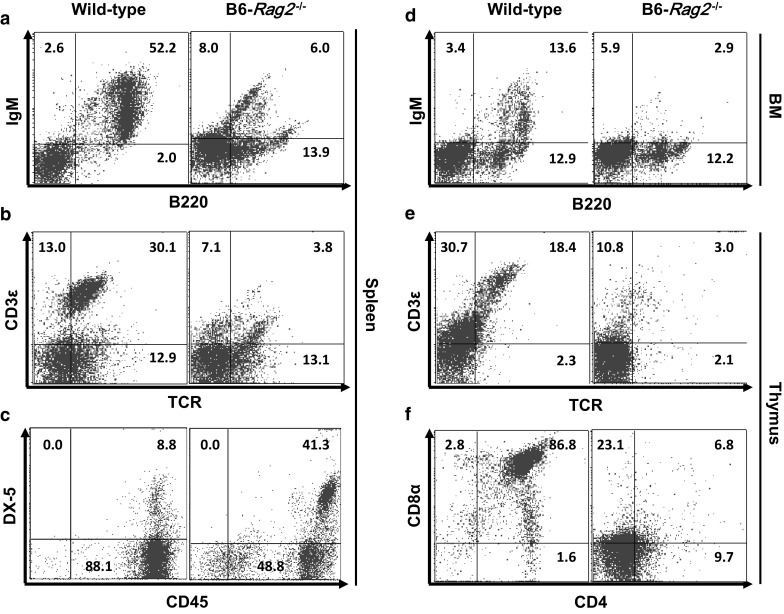
Flow cytometric analysis of lymphoid cells in 8-week-old B6-*Rag2*^−*/*−^ mice. **a**–**c** analysis of B, T, and NK cells in the spleen. **d** Analysis of B cells in the bone marrow. **e**, **f** Analysis of T cells in the thymus. Each value represents the mean ± SD. (n = 10 mice per group)

Gross examination of *Rag2*^−/−^ mice (both FVB and B6) revealed that its spleen and thymus were smaller than those of wild-type mice. Furthermore, the spleen-to-body weight ratios of both types and both sexes of *Rag2*^−*/*−^ mice were significantly lower than that of wild-type mice (*P* < 0.05; Fig. 1). Flow cytometric analyses showed a markedly decreased population of mature B cells, especially B220^+^IgM^+^ cells in the spleen (*P* < 0.001; Figs. 2a, 3a), suggesting that Rag2 deficiency leads to impaired B cell differentiation (Shinkai et al. 1992). Both FVB-*Rag2*^−/−^ and B6-*Rag2*^−/−^ mice exhibited a significantly decreased number of B220^+^IgM^+^ B cells in the bone marrow where B lymphocytes mature (*P* < 0.001; Figs. 2d, 3d).

Early T cells differentiate from CD4^+^CD8^+^ double-positive T cells into CD4^+^ or CD8^+^ single-positive mature T cells, with the latter also expressing T-cell receptors (TCR) and the CD3 complex (Germain 2002). The proportion of CD4^+^CD8α^+^ double-positive thymocytes, mature CD4^+^ or CD8α^+^ single-positive thymocytes, and TCR^+^CD3ε^+^ double-positive mature thymocytes was markedly decreased in both *Rag2*-deficient mouse strains. Interestingly, B6-*Rag2*^−/−^ mice (6.8%; Fig. 3f) exhibited slightly higher populations of CD4^+^CD8α^+^ double-positive thymocytes than FVB-*Rag2*^−*/*−^ mice (1.9%; Fig. 2f). Although the ratio of TCR^+^CD3ε^+^ double-positive cells in the spleen was higher in wild-type B6 mice (30.1%) than that in wild-type FVB mice (17.7%), both FVB-*Rag2*^−*/*−^ and B6-*Rag2*^−*/*−^ mice exhibited similar decreases in the TCR^+^CD3ε^+^ double-positive thymocyte population compared to wild-type mice.

The NK cell population was confirmed by examination of CD45^+^DX-5^+^ marker expression in *Rag2*^−/−^ mice. Compared to the wild-type, CD45^+^DX-5^+^ double-positive NK cells in the spleen were significantly increased by more than fourfold in both FVB-*Rag2*^−/−^ (wild-type, 10.4%; homozygous null, 41.9%) and B6-*Rag2*^−*/*−^ mice (wild-type, 8.8%; homozygous null, 41.3%) (*P* < 0.001; Figs. 2c, 3c).

### Immunophenotypes of *Il2rg*^−*/*−^ mice

**Fig. 4:**
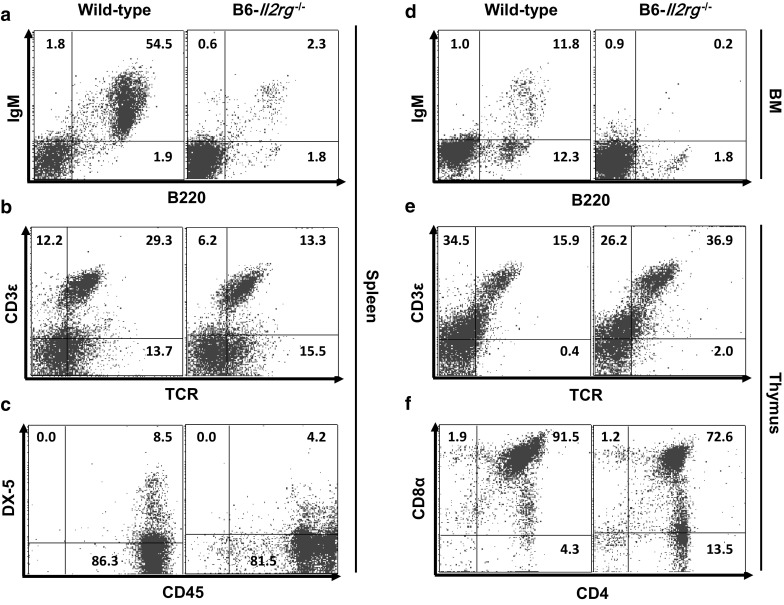
Flow cytometric analysis of lymphoid cells in 8-week-old B6-*Il2rg*^−*/*−^ mice. **a**–**c** analysis of B, T, and NK cells in the spleen. **d** Analysis of B cells in the bone marrow. **e**, **f** Analysis of T cells in the thymus. Each value represents the mean ± SD. (n = 10 mice per group)

The spleen-to-body weight ratio of both sexes of B6-*Il2rg*^−*/*−^ mice was greatly increased compared to that of wild-type mice (*P* < 0.01; Fig. 1). Similar to *Rag2*^−*/*−^ mice, immunophenotyping analysis of B lymphocytes in the spleen revealed a sharp reduction in the number of B220^+^IgM^+^ double-positive B lymphocytes (*P* < 0.001; Fig. 4a). B6-*Il2rg*^−*/*−^ mice also displayed a lack of functional mature B220^+^IgM^+^ B lymphocytes in the bone marrow (Fig. 4d).

Unlike the immunophenotyping results of *Rag2*^−*/*−^ mice which confirmed TCR^+^CD3ε^+^ double-positive T cell deficits, a group of remnant T lymphocytes were observed in B6-*Il2rg*^−*/*−^ mice (wild-type, 29.3%; homozygous null, 13.3%, Fig. 4b). Not only were T cells found in the spleen, but a large number of CD4^+^CD8α^+^ double-positive T lymphocytes were located in the thymus as well (wild-type, 91.5%; homozygous null, 72.6%; Fig. 4f). Interestingly, the number of TCR^+^CD3ε^+^ double-positive T thymocytes was increased by more than twofold when compared to those in the wild-type (wild-type, 15.9%; homozygous null, 36.9%; Fig. 4e).

The cell population of CD45^+^DX-5^+^ double-positive NK cells in B6-*Il2rg*^−*/*−^ mice was decreased by 50% in the spleen (*P* < 0.05; wild-type, 8.5%; homozygous null, 4.2%; Fig. 4c).

### Immunophenotypes of *Prkdc*^−/−^ mice

**Fig. 5:**
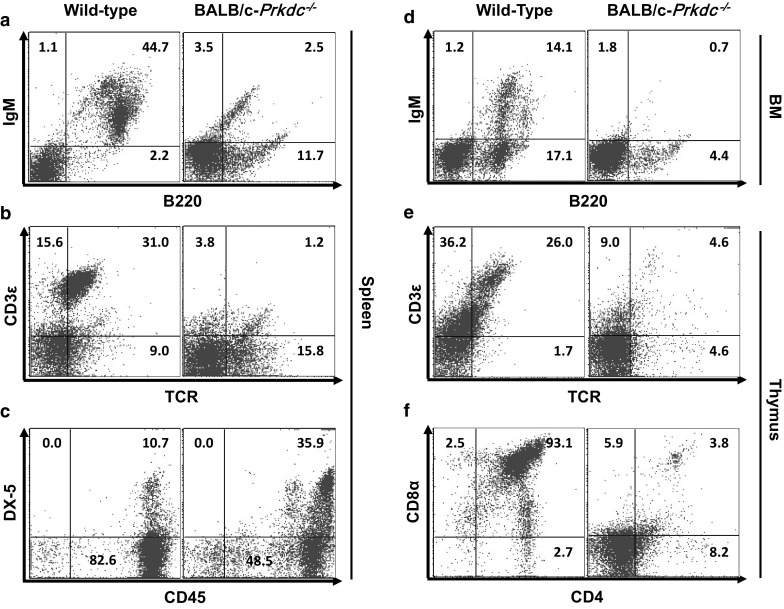
Flow cytometric analysis of lymphoid cells in 8-week-old BALB/c-*Prkdc*^−/−^ mice. **a**–**c** analysis of B, T, and NK cells in the spleen. **d** Analysis of B cells in the bone marrow. **e**, **f** Analysis of T cells in the thymus. Each value represents the mean ± SD. (n = 8–10 mice per group)

The spleen and thymus in BALB/c-*Prkdc*^−*/*−^ mice were smaller than those of wild-type mice. Furthermore, the spleen-to-body weight ratio of female, but not male, BALB/c-*Prkdc*^−*/*−^ mice was much smaller than that of wild-type mice (*P* < 0.001; Fig. 1). Flow cytometric analysis showed marked differences between homozygous null and wild-type mice: populations of both B220^+^IgM^+^ mature B lymphocytes in the spleen and bone marrow and TCR^+^CD3ε^+^ double-positive T lymphocytes in the thymus and spleen were significantly decreased (*P* < 0.001; Fig. 5a–b, d–e). CD4^+^CD8α^+^ double-positive T lymphocytes could barely be detected in the thymus (Fig. 5f).

Additionally, there was a threefold increase in the number of CD45^+^DX-5^+^ double-positive spleen NK cells in BALB/c-*Prkdc*^−*/*−^ mice (36.0%) compared to that in wild-type mice (10.7%; *P* < 0.001; Fig. 5c).

### Serum immunoglobulin levels

**Fig. 6:**
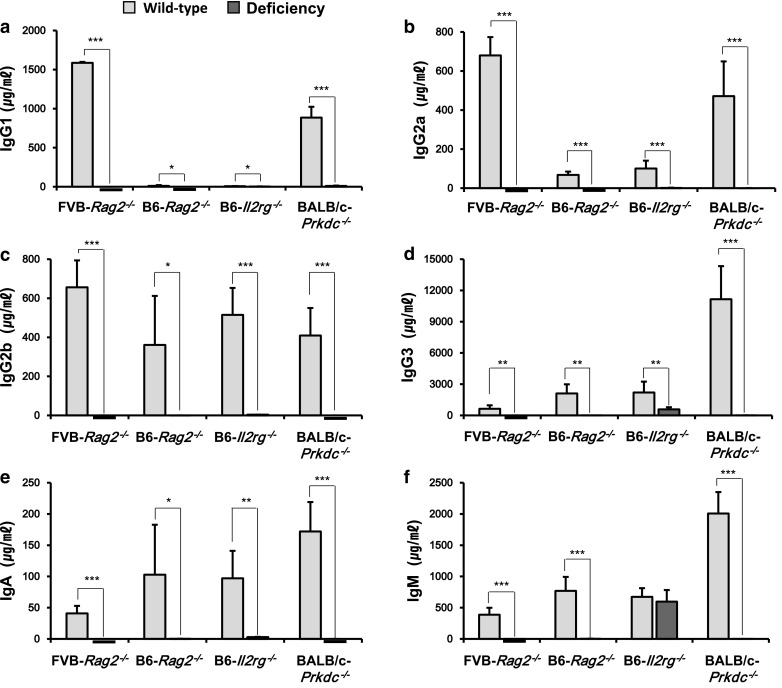
Levels of serum immunoglobulins in FVB-*Rag2*^−/−^, B6-*Rag2*^−/−^, B6-*Il2rg*^−/−^, and BALB/c-*Prkdc*^−/−^ mice. The serum levels of IgG1 (**a**), IgG2a (**b**), IgG2b (**c**), IgG3 (**d**), IgA (**e**), and IgM (**f**) were measured by ELISA. Each value represents the mean ± SD. (n = 8–10 mice per group). *P < 0.05, **P < 0.01, and ***P < 0.001

Because immunodeficient mice are defective in B cell maturation, the serum immunoglobulin levels of IgG1, IgG2a, IgG2b, IgG3, IgA, and IgM were assessed by ELISA in our four immunodeficient mouse strains (Fig. 6). All immunoglobulin levels in FVB-*Rag2*^−/−^, B6-*Rag2*^−/−^, and BALB/c-*Prkdc*^−/−^ mice were significantly decreased, particularly those in FVB-*Rag2*^−/−^ mice, which were hardly detectable (*P* < 0.05 to *P* < 0.001). Interestingly, the IgM level was not significantly changed in B6-*Il2rg*^−*/*−^ mice (wild type, 674 μg/μL; homozygote, 597.8 μg/μL; Fig. 6f), and all immunoglobulin levels in B6-*Il2rg*^−*/*−^ mice were the highest among those in the four immunodeficient mouse strains (IgG1, 3.6 μg/μL; IgG2a, 1.4 μg/μL; IgG2b, 3.7 μg/μL; IgG3, 586.8 μg/μL; IgA, 2.7 μg/μL; IgM, 597.8 μg/μL).

### Immunohistochemical findings

Immunohistochemical analysis of organs from the two *Rag2*-deficient mouse strains by flow cytometry and ELISA demonstrated that they had severe deficits in B and T lymphocytes. B220 staining of the spleen and lymph nodes of *Rag2*^−/−^ mice showed a considerable reduction in the number of positive cells (Fig. S3). Splenic nodular hypoplasia was observed in both *Rag2*^−/−^ mice, and a noticeable decrease in the number of CD4^+^ T cells was observed in the spleen, lymph nodes, and thymus (Fig. S3). However, the number of residual B220^+^ cells was slightly higher in B6-*Rag2*^−/−^ mice than in FVB-*Rag2*^−*/*−^ mice.

## Discussion

Numerous immunodeficient mouse models have been generated for biomedical research involving engraftment and transplantation of hematopoietic cells and xenografting of tumor cells or tissues (Greiner et al. 1998; Majeti et al. 2007; Shultz et al. 2007). We developed four immunodeficient mouse models using CRISPR/Cas9 and analyzed the immunophenotype of each homozygous null mouse to determine the degree of immune deficiency, particularly the levels of B, T, and NK cells and immunoglobulins. Our FVB-*Rag2*^−/−^ (*Rag2*^*em1Hwl*^), B6-*Rag2*^−/−^(*Rag2*^*em2Hwl*^), and BALB/c-*Prkdc*^−/−^ (*Prkdc*^*em1Hwl*^) mice exhibited phenotypes slightly different from those of conventional models (Table S3). *Rag2*-deficient mice generated in 1992 were reported to have a smaller thymus but spleen size was not affected (Shinkai et al. 1992). In contrast, our *Rag2*^−/−^ mice showed both smaller spleen and thymus. Furthermore, we found that both *Rag2*^−/−^ and *Prkdc*^−/−^ mice lacked mature B and T cells: the numbers of B220^+^IgM^+^ mature B cells in the spleen and bone marrow and TCR^+^CD3ε^+^ mature T cells in the spleen and thymus were decreased dramatically in homozygous null mice. Serum immunoglobulin levels in B6-*Rag2*^−/−^ and BALB/c-*Prkdc*^−*/*−^ mice were undetectable, particularly in FVB-*Rag2*^−/−^ mice. Flow cytometry and serum immunoglobulin levels showed that B6-*Rag2*^−/−^ mice had a slightly increased leakage compared to FVB-*Rag2*^−/−^ or BALB/c-*Prkdc*^−/−^ mice. This leakiness varies by background strain and age (Nonoyama et al. 1993). Consistent with these findings, effector CD4 T cell differentiation was enhanced in the mesenteric lymph nodes of B10-*Rag2*^−/−^ compared to that in our B6-*Rag2*^−/−^ mice (Valatas et al. 2013).

Distinctively, our B6-*Il2rg*^−*/*−^ (*Il2rg*^*em1Hwl*^) mice exhibited phenotypes different from those of conventional *Il2rg*-deficient mouse models (Table S3), in particular demonstrating much more abundant presence of mature T cells. The number of TCR^+^CD3ε^+^ T lymphocytes was decreased in the spleen of homozygote knockout mice, but there was a 2.3-fold increase in the number of TCR^+^CD3ε^+^ mature thymocytes. Interestingly, more CD4^+^CD8α^+^ thymocytes remained in B6-*Il2rg*^−*/*−^ mice compared to those in both *Rag2*^−/−^ and *Prkdc*^−*/*−^ mice. Serum immunoglobulin levels in B6-*Il2rg*^−*/*−^ mice were dramatically decreased, except for that of IgM. Similar to our findings, an X-SCID rat model showed total IgG and IgA levels that were severely decreased except for the IgM level (Mashimo et al. 2010). In fact, the serum IgM level was found to be higher in *Il2rg*^−*/*−^ mice than in control wild-type mice, although the number of CD45R^+^sIgM^+^ B cells was severely decreased as reported (Ohbo et al. 1996).

We found that the number of CD45^+^DX-5^+^ NK cells in B6-*Il2rg*^−*/*−^ mice was twofold lower than that in wild-type mice and confirmed that TCRαβ^−^NK1.1^+^ NK cells were not detectable in the spleen of *Il2rg*^−/−^ mice (Ohbo et al. 1996). However, the C.B-17 SCID mouse reported in 1983 exhibited unaffected NK cell function (Bosma et al. 1983; Dorshkind et al. 1985). Consistent with previous reports, all of the homozygous *Rag2* and *Prkdc* knockout mice generated in this study expressed three- to four-fold elevated CD45^+^DX-5^+^ NK cell populations compared to wild-type mice. These results may reflect immune compensation for the lack of mature B and T lymphocytes and should be further examined to determine the accurate functions of the *Rag2*, *Prkdc*, and *Il2rg* genes.

In summary, our newly generated immunodeficient mice exhibited some differences from conventional immunodeficient mouse strains, particularly the B6-*Il2rg*^−*/*−^ mice. The homozygous FVB-*Rag2*^−/−^, B6-*Rag2*^−/−^, and BALB/c-*Prkdc*^−/−^ mice showed similar phenotypes including a lack of mature B cells and mature T cells as well as similar serum immunoglobulin levels. However, B6-*Il2rg*^−*/*−^ mice showed unique phenotypes, lacking mature B cells and possessing increased mature T cell numbers and high serum IgM levels. The NK cell population in B6-*Il2rg*^−*/*−^ mice was also decreased. The difference between our mutant strains and the conventional immunodeficient mouse models may be due to the difference in the production methods. The conventional models generated by ES cell-based gene targeting contain a drug resistance marker such as a PGK-neo cassette in order to enable screening of correctly targeted clones. Thus, expression of the neomycin-resistance gene can have an effect on gene expression and cell physiology (Scacheri et al. 2001; Valera et al. 1994). On the other hand, our models generated using the CRISPR/Cas9 system lacked any insertion of exogenous DNA sequences. Therefore, these immunodeficient mouse models may be a useful tool for gaining further insight into the immune system. We can consider using our mouse models to improve current humanized mouse models such as the NOD SCID gamma (NSG) mice that are being vastly utilized presently at the cutting edge of this field.

## Electronic supplementary material

Below is the link to the electronic supplementary material.

Supplementary material 1 (DOCX 2738 kb)
