# Supplementary material for: Differences between immunodeficient mice generated by classical gene targeting and CRISPR/Cas9-mediated gene knockout
Source: Transgenic Res. 2018 Mar 28;27(3):241–51. doi: 10.1007/s11248-018-0069-y (PMC5986857; doi:10.1007/s11248-018-0069-y)
Supplement: Supplementary file 1 — Supplementary material 1 (DOCX 2738 kb) [file 11248_2018_69_MOESM1_ESM.docx]

**Differences between immunodeficient mice generated by classical gene targeting and CRISPR/Cas9-mediated gene knockout**

Jae Hoon Lee^1,3^, Jong-Hyung Park^2,3^, Tae-Wook Nam^1^, Sun-Min Seo^2^, Jun-Young Kim^2^, Han-Kyul Lee^2^, Jong Hyun Han^1^, Song Yi Park^1^, Yang-Kyu Choi^2^*, and Han-Woong Lee^1^*

^1^Department of Biochemistry, College of Life Science and Biotechnology, Laboratory Animal Research Center, Yonsei University, Seoul 03722, Republic of Korea

^2^Department of Laboratory Animal Medicine, College of Veterinary Medicine, Konkuk University, Seoul 05029, Republic of Korea

^3^These authors contributed equally to this work.

*Corresponding authors:

Department of Biochemistry, College of Life Science and Biotechnology, Yonsei University, Seoul 03722, Republic of Korea

Phone: 82-2-2123-5698 Fax: 82-2-2123-8107

E-mail: hwl@yonsei.ac.kr

Department of Laboratory Animal Medicine, College of Veterinary Medicine, Konkuk University, Seoul 05029, Republic of Korea

Phone: 82-2-2049-6113 Fax: 82-2-450-3037

E-mail: yangkyuc@konkuk.ac.kr

**Fig. S1. CRISPR/Cas9-mediated gene-targeting strategy to generate immunodeficient mice.**

(a, b) Schematics showing the locus and sequences of *Rag2* alleles in the FVB-*Rag2*^-/-^ (a) and B6-*Rag2^-/-^* (b) mouse. (b) The upper band (Up) in the gel image is amplified between primers *Rag2* WT F and *Rag2* KO R (Table S2) that were designed in the flanking region of the mutation site.

(c) Schematics showing the locus and sequences of the *Il2rg* alleles in the B6-*Il2rg^-/-^* mouse are displayed.

(d) CRISPR/Cas9 targeting schematics of the *Prkdc* locus of chromosome 16. Both wild-type and knockout sequences in exons 3 and 41 after CRISPR/Cas9 excision are displayed.

The initiation codon is labeled green, the guide sequence of each sgRNA is indicated in blue, and the protospacer-adjacent motif (PAM) is in red. The stop codon is indicated by a red asterisk (*). Right panels of each targeting schematic shows the genotyping result by PCR using genomic DNA for the presence of the KO (deleted) allele.

**
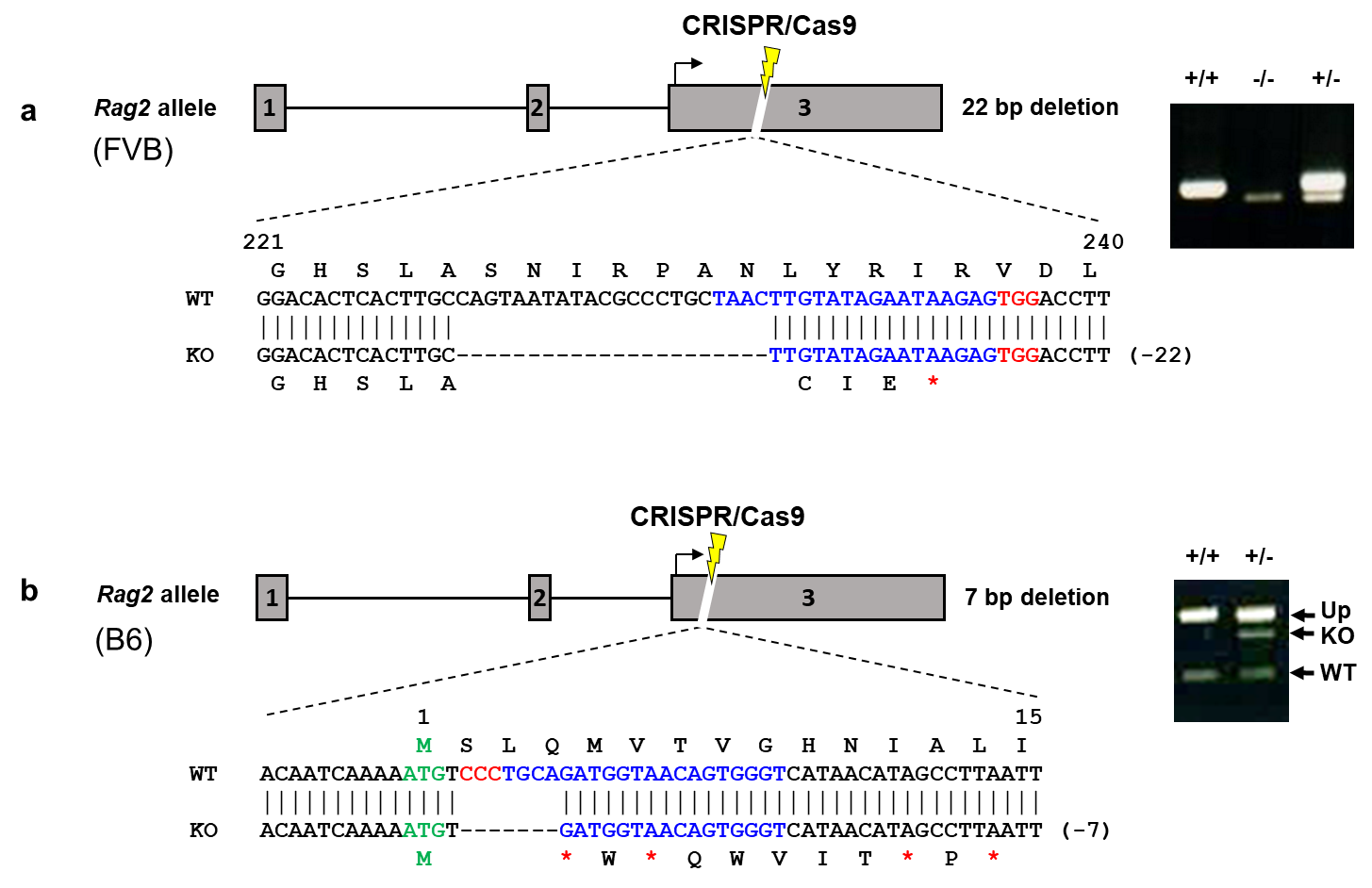
**

**Fig. S1 (Continued)**

**
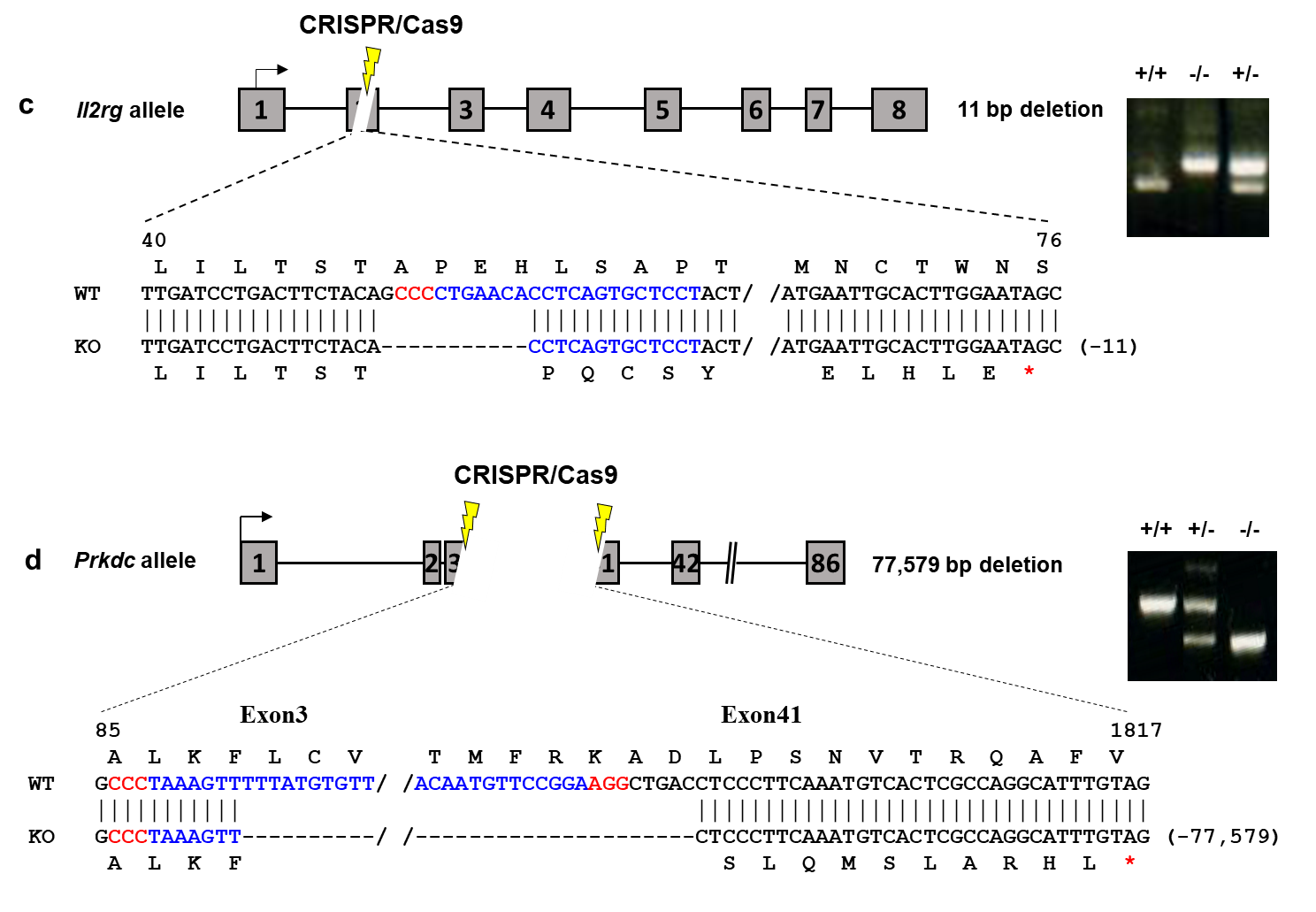
**

**Fig. S2. The mRNA expression in the immunodeficient mice using CRISPR/Cas9-mediated gene-targeting.**

The expression of mRNAs encoding each target gene was analyzed in the immune system tissues of FVB-*Rag2*^-/-^ (a), B6-*Rag2*^-/-^ (b), B6-*Il2rg*^-/-^ (c), and BALB/c-*Prkdc*^-/-^ (d) mouse by RT-PCR. *Gapdh* was used as an internal control.

**
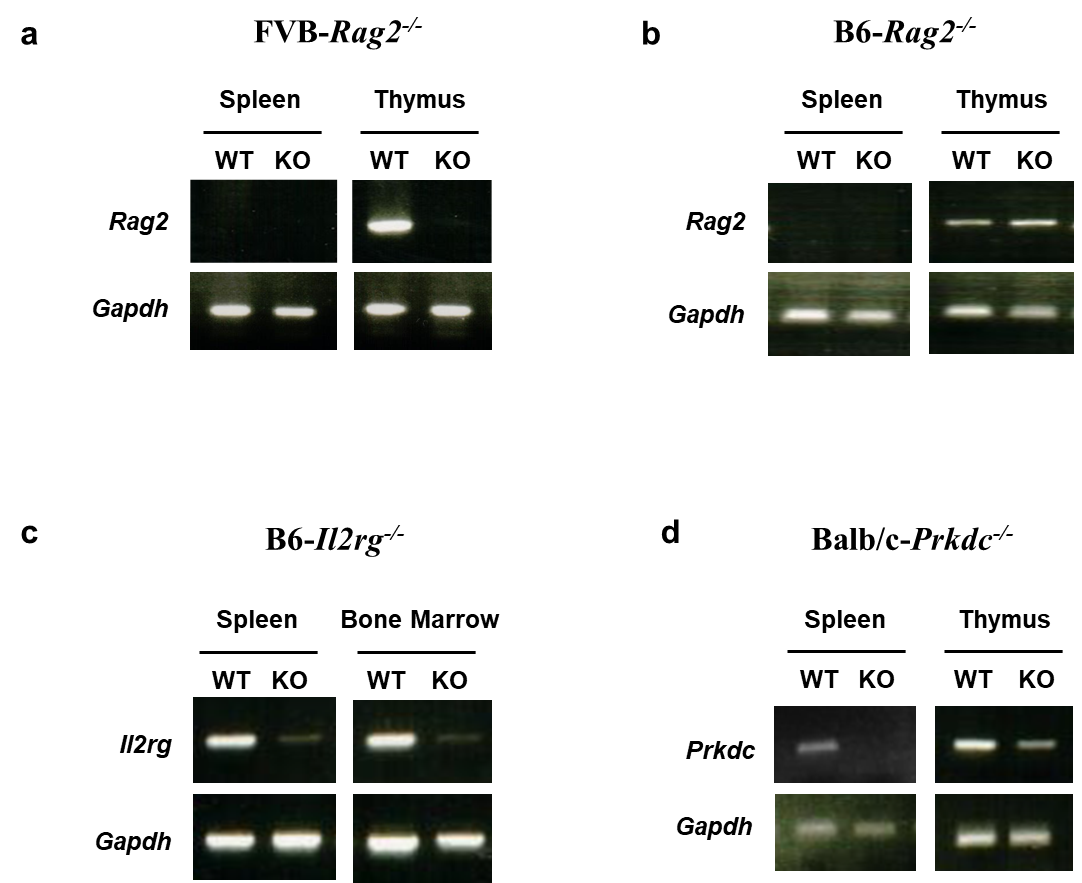
**

**Fig. S3. Immunohistochemical staining for B220 and CD4 in the spleen, lymph node, and thymus of 8-week-old *Rag2^-/-^* mice.**

(Upper row) B220^+^ cells in the spleen; (Middle row) CD4^+^ cells in the lymph node; (Bottom row) CD4^+^ cells in the thymus.

**
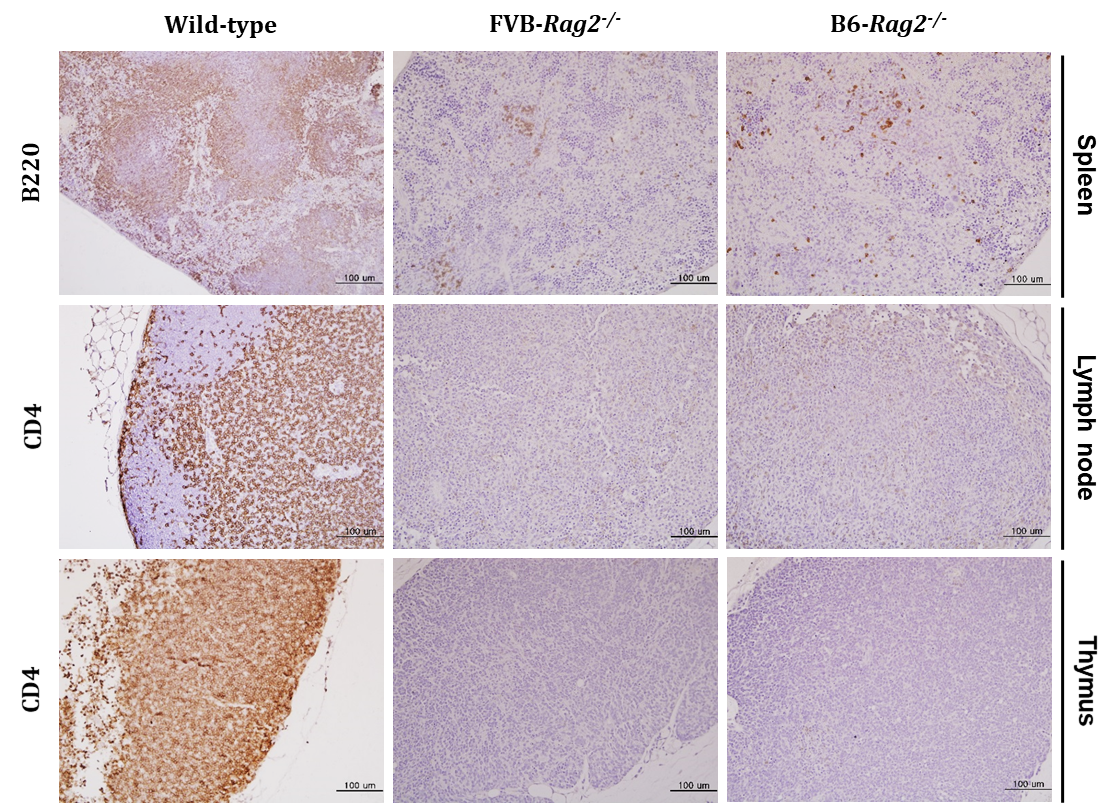
**

**Table S1. CRISPR/Cas9-mediated gene targeting in one-cell mouse embryos.**

| **Target Gene** | **Mouse strain** | **Cas9 mRNA (ng/μL)** | **Total sgRNA  (ng/μL)** | **No. of sgRNAs** | **No. of embryos injected** | **No. of embryos transfered^b^ (%)** | **No. of newborns (%)** | **No. of mutants (%)** |
| --- | --- | --- | --- | --- | --- | --- | --- | --- |
| *Rag2* | FVB | 50 | 250 | 1 | 129 | 120 (93) | 16 (13) | 4 (25) |
| *Rag2* | B6J | 50 | 200 | 1 | 102 | 70 (69) | 21 (30) | 3 (14) |
| *Il2rg* | B6J | 50 | 250 | 1 | 251 | 194 (77) | 42 (22) | 13 (31) |
| *Prkdc* | Balb/c | 20 | 200^a^ | 4 | 356 | 236 (66) | 45 (33) | 37^c^ (82) |
| a. Each sgRNA was used at 50 ng/μL.  b. All embryos that survived after the microinjection were transferred into the oviducts of foster mothers. c. Large deletions were found in 9 mice (24%), and small indels in 28 mice (75%). | | | | | | | | |

**Table S2. PCR primers used to genotype immunodeficient mouse strains.**

| **Primer set** | **Primer sequence (5’ - 3’)** | **Product length (bp)** |
| --- | --- | --- |
| **FVB-*Rag2*** | | |
| *Rag2* FVB F | CTCCCAGAACTTCAGGATGGGCT | 330 (WT)  305 (KO) |
| *Rag2* FVB R | AGTCAGGAGTCTCCATCTCACTGA |  |
| **B6-*Rag2*** | | |
| *Rag2* WT F (WT) | ACAATCAAAAATGTCCCTGCA | 65 (WT) |
| *Rag2* WT R | AGCCTGGTTGAATTAAGGCTATG |  |
| *Rag2* KO F | GCTGCTGCCACAATAAAGTAGTG | 230 (KO) |
| *Rag2* KO R (KO) | CCACTGTTACCATC ACATTT |  |
| **B6-*Il2r****γ* | | |
| *Il2rg* WT F (WT) | GACTTCTACAGCCCCTGA | 200 (WT) |
| *Il2rg* WT R | CTCTCCCAGCTAACCTCCCT |  |
| *Il2rg* KO F | GTCCTCATGTCCAGTGCGAA | 258 (KO) |
| *Il2rg* KO R (KO) | GGAGCACTGAGGTGT AG |  |
| **BALB/c-*Prkdc*** | | |
| *Prkdc* F | CCCCAGCATTGCAGATTTCC |  |
| *Prkdc* WT | GAAAGTGACTGCTGGATGGC | 427 (WT) |
| *Prkdc* KO | TCACAGTGCCACAACAAGGT | 277 (KO) |
| The primers Rag2 WT F and Il2rg WT F are annealed to the sequence of WT allele of each gene, whereas Rag2 KO R and Il2rg KO R to the sequence of knockout allele of each gene. Red fonts indicate the WT-specific regions (the deleted sequences in the knockout alleles) of primers. | | |

Table S3. Phenotypic comparison of immunodeficient mice^a^.

|  | Background strain | Targeting strategy | Serum IgM level | Mature  T cell population | Mature  B cell population | NK cell  population | Weight of spleen | Reference |
| --- | --- | --- | --- | --- | --- | --- | --- | --- |
| FVB-*Rag2^em1Hwl^* | FVB | CRISPR/Cas9 | Undetectable | Decreased  (⨯0.2) | Decreased  (⨯0.1) | Increased  (⨯4) | Decreased  (⨯0.45) |  |
| B6-*Rag2^em2Hwl^* | B6 | CRISPR/Cas9 | Undetectable | Decreased  (⨯0.13) | Decreased  (⨯0.11) | Increased  (⨯5) | Decreased  (⨯0.66) |  |
| B6;129-*Rag2^tm1Fwa^* | B6;129/Sv | ES cells | Undetectable | Undetectable | Undetectable | Intact | Decreased | (Shinkai et al., 1992) |
| BALB/c-*Prkdc^em1Hwl^* | BALB/c | CRISPR/Cas9 | Undetectable | Decreased  (⨯0.04) | Decreased  (⨯0.06) | Increased  (⨯3) | Decreased  (⨯0.63) |  |
| C.B17-*Prkdc^scid^* | C.B17 | Spontaneous Mutation | Undetectable | Undetectable | Undetectable | Intact | Decreased | (Shinkai et al., 1992)  (Shultz et al., 2007) |
| B6-*Il2rg^em1Hwl^* | B6 | CRISPR/Cas9 | Intact | Increased  (⨯2) | Decreased  (⨯0.04) | Decreased  (⨯0.5) | Increased  (⨯3.5) |  |
| B6;129-*Il2rg^tm1Wjl^* | B6;129/Sv | ES cells | Increased  (⨯3) | Decreased  (⨯1/10) | Decreased  (⨯0.25) | Undetectable | Increased  (⨯2.0) | (Ohbo et al., 1996)  (DiSanto et al., 1995) |

^a^ Each strain is compared to wild-type mice.

DiSanto, J.P., Muller, W., Guy-Grand, D., Fischer, A., and Rajewsky, K. (1995). Lymphoid development in mice with a targeted deletion of the interleukin 2 receptor gamma chain. Proc Natl Acad Sci U S A *92*, 377-381.

Ohbo, K., Suda, T., Hashiyama, M., Mantani, A., Ikebe, M., Miyakawa, K., Moriyama, M., Nakamura, M., Katsuki, M., Takahashi, K.*, et al.* (1996). Modulation of hematopoiesis in mice with a truncated mutant of the interleukin-2 receptor gamma chain. Blood *87*, 956-967.

Shinkai, Y., Rathbun, G., Lam, K.P., Oltz, E.M., Stewart, V., Mendelsohn, M., Charron, J., Datta, M., Young, F., Stall, A.M.*, et al.* (1992). RAG-2-deficient mice lack mature lymphocytes owing to inability to initiate V(D)J rearrangement. Cell *68*, 855-867.

Shultz, L.D., Ishikawa, F., and Greiner, D.L. (2007). Humanized mice in translational biomedical research. Nat Rev Immunol *7*, 118-130.
